# Trastuzumab Blocks the Receiver Function of HER2 Leading to the Population Shifts of HER2-Containing Homodimers and Heterodimers

**DOI:** 10.3390/antib10010007

**Published:** 2021-02-04

**Authors:** Jun Zhao, Nishant Mohan, Ruth Nussinov, Buyong Ma, Wen Jin Wu

**Affiliations:** 1 Division of Biotechnology Review and Research 1, Office of Biotechnology Products, Office of Pharmaceutical Quality, Center for Drug Evaluation and Research, U.S. Food and Drug Administration, 10903 New Hampshire Avenue, Silver Spring, MD 20993, USA; Nishant.Mohan@fda.hhs.gov; 2 Interagency Oncology Task Force (IOTF) Fellowship: Oncology Product Research/Review Fellow, National Cancer Institute, Bethesda, MD 20892, USA; 3 Basic Science Program, Leidos Biomedical Research, Inc., Cancer and Inflammation Program, National Cancer Institute, Frederick, MD 21702, USA; nussinor@mail.nih.gov (R.N.);

**Keywords:** trastuzumab, monoclonal antibody, epidermal growth factor (EGF), human epidermal growth factor receptor 2 (HER2), HER2-positive breast cancer, monomer, homodimer, heterodimer, antagonist, agonist, simulation

## Abstract

HER2, a member of the Erythroblastosis Protein B/Human Epidermal Growth Factor Receptor (ErbB/HER) family of receptor tyrosine kinase, is overexpressed in 20~30% of human breast cancers. Trastuzumab, a HER2-targeted therapeutic monoclonal antibody, was developed to interfere with the homodimerization of HER2 in HER2-overexpressing breast cancer cells, which attenuates HER2-mediated signaling. Trastuzumab binds to the domain IV of the HER2 extracellular domain and does not directly block the dimerization interface of HER2-HER2 molecules. The three-dimensional structures of the tyrosine kinase domains of ErbB/HER family receptors show asymmetrical packing of the two monomers with distinct conformations. One monomer functions as an activator, whereas the other acts as a receiver. Once activated, the receiver monomer phosphorylates the activator or other proteins. Interestingly, in our previous work, we found that the binding of trastuzumab induced phosphorylation of HER2 with the phosphorylation pattern of HER2 that is different from that mediated by epidermal growth factor (EGF) in human epidermal growth factor receptor 2 (HER2)-positive breast cancer. Binding of trastuzumab to HER2 promoted an allosteric effect of HER2, in both tyrosine kinase domain and ectodomain of HER2 although details of allosteric regulation were missing. In this study, we utilized molecular dynamics (MD) simulations to model the allosteric consequences of trastuzumab binding to HER2 homodimers and heterodimers, along with the apo forms as controls. We focused on the conformational changes of HER2 in its monomeric and dimeric forms. The data indicated the apparent dual role of trastuzumab as an antagonist and an agonist. The molecular details of the simulation provide an atomic level description and molecular insight into the action of HER2-targeted antibody therapeutics.

## 1. Introduction

The ErbB family of receptor tyrosine kinases, including ErbB1/EGFR/HER1, ErbB2/HER2, ErbB3/HER3, and ErbB4/HER4, share common structural features. They consist of a ligand-binding extracellular domain and an intracellular domain connected by a single transmembrane helix. The extracellular domain consists of four domains, I-IV, and the intracellular domain consists of a short juxtamembrane segment, a tyrosine kinase domain, and a long unstructured C-terminal tail. The activation of EGFR depends on EGF-induced receptor dimerization and the structural arrangement of the ligand-induced dimers [1,2]. In this arrangement, the extracellular domains form a back-to-back dimer [3,4] with the two-ligand-binding sites distal to the dimer interface. Dimerization of the extracellular domains, by conformational coupling across the membrane [5,6] promotes formation of catalytically active asymmetric kinase dimers [7] that auto-phosphorylate the C-terminal tails and initiate downstream signaling. In this asymmetrical interface, the C-terminal lobe (αH) of one kinase (activator) interacts with the N-terminal lobe (αC) of the other kinase (receiver). In the case of the HER3-HER2 heterodimer, although the αC of HER3 is different from other HER family members, the αH is conserved. Thus, the C-terminal lobe of HER3 can still activate the tyrosine kinase domain of HER2 [8,9]. HER3 and HER4 have similar activation mechanisms as EGFR, though the ligand specific to HER2 has not yet been identified. The HER2 extracellular domain adopts a fixed conformation, which is similar to the ligand-activated state of EGFR that allows HER2 to form homo- or heterodimers with other HER family receptors in the absence of a ligand [10,11].

Trastuzumab is a monoclonal antibody specifically designed to target the extracellular domain IV of HER2. The mechanisms of actions of trastuzumab include antibody dependent cellular cytotoxicity (ADCC), antibody-mediated HER2 internalization followed by receptor degradation, inhibition of HER2 dimerization, and protection of the HER2 extracellular domain from proteolysis [12]. Therapeutic binding of monoclonal antibodies to a cell surface receptor may elicit either an agonistic or antagonistic effect on the receptor. As an antagonist, the antibodies block or dampen a biological response mediated by the receptors. As an agonist, the antibodies bind the receptor in a manner that mimics the binding of the natural ligand. Our results indicate that when HER2 functions as a receiver, trastuzumab inhibits dimerization as an antagonist between HER2 and other HER family receptors. When HER2 functions as activator, trastuzumab does not block dimerization of HER2 and other ErbB family proteins, but it induces structural changes in the tyrosine kinase domain as an agonist. We found that, in vitro, trastuzumab binding leads to changes in the phosphorylation site compared to ligand-dependent phosphorylation. These data indicate the dual role of trastuzumab as an agonist and an antagonist.

## 2. Materials

### 2.1. Molecular Modeling and Simulations

The sequence of HER2 was obtained from UniProt.org (identifier: P04626-1, isoform 1). First, homology modeling was used to obtain the different domains of HER2 separately. Domains I, II, and III of the ectodomain dimer were built based on the structure of the HER2 dimer [13] obtained from long MD simulation. Domain IV of the ectodomain dimer was built based on the structure of the EGFR dimer in the bound state [5] obtained from long MD simulation. The transmembrane and juxtamembrane domains were built based on the NMR structure of EGFR (PDBID: 2M20 [6]). The tyrosine kinase domain was built based on the crystal structure (PDBID: 3PP0 [14]). The homology structures of the domains were superimposed on the bound state EGFR dimer [5] to obtain the overall structure of the HER2 dimer. The kinase domains of HER2-HER1 and HER2-HER3 heterodimers were built based on 2GS6 [7] and 4RIW [15] from the Protein Data Bank, respectively. The ectodomain dimer, transmembrane domain, and the juxtamembrane domain were built based on the EGFR dimer [5] obtained from long MD simulation. The HER2 dimer-trastuzumab complex was constructed by superimposing the 1N8Z [10] structure on the HER2 dimer structure. HER2 monomer was obtained by removing one monomer from HER2 dimer. The HER2 monomer-trastuzumab complex, HER2 monomer-pertuzumab complex, and HER2 monomer-trastuzumab-pertuzumab complex were constructed by superimposing the 1N8Z, 1S78 [16], and both of 1N8Z/1S78 structures on the HER2 monomer structure, respectively. Trastuzumab and pertuzumab Fabs structures are built based on 1S78 and 1N8Z, respectively.

The conserved disulfide bonds of HER2 and the antibody Fabs were constructed according to UniProt and the PDB files of trastuzumab and pertuzumab. As the non-sequential Kabat numbering scheme is used in the crystal structures of Fabs, the residues were renumbered for convenience in the simulation. The N- and C-termini were charged as NH_3_^+^ and COO^−^ groups, respectively. HER2 Tyr877 was phosphorylated. The above HER2 related complexes were inserted into a POPC/POPS bilayer using the CHARMM-GUI membrane builder generator [17]. The lipid content was 30% POPS and 70% POPC in the intracellular and 100% POPC in the extracellular leaflets. The lipid composition mimics the mammalian plasma membrane in which ~15% lipids in total are anionic, which are almost exclusively found in the intracellular leaflet of cell membranes.

The systems were then solvated by TIP3P water molecules, and sodium and chlorides were added to neutralize the system and to achieve a total concentration of ~150 mM. The systems were minimized for 5000 conjugate gradient steps, with the protein backbone atoms and lipid head group atoms fixed followed by an additional 5000 conjugate gradient minimization steps that were applied to the systems. In the equilibration run, each system was gradually relaxed by a series of dynamic cycles, in which the harmonic restraints on the proteins and lipid head groups were gradually released to optimize the protein-water, protein-lipid, and water-lipid interactions. In the production stage, NPAT (constant number of atoms, pressure, surface area, and temperature) ensemble at 310 K was used. The surface area in the *xy* plane (membrane plane) remained constant with a volume change in the *z* direction. A switch function with a twin-range cutoff at 12 and 14 Å was used to evaluate the van der Waals (vdW) interactions. The Particle Mesh Ewald (PME) method was used for long-range electrostatic interactions evaluation. Each simulation system was run twice using the same strand configuration, with different lipid conformations randomly selected from the lipid library, and different initial velocities for all atoms. All MD simulations were performed using NAMD software [18] with a CHARMM36 force field. MD trajectories were saved every 2 ps for analysis.

### 2.2. Potential Energy Evaluation

To evaluate the total potential energy of the system, the trajectory for each system was extracted from the last 20 ns of explicit solvent MD without water molecules and ions. The solvation energies of all systems were calculated using the generalized Born method with molecular volume (GBMV) [19] after 500 steps of energy minimization to relax the local geometries caused by the thermal fluctuations which occurred in the MD simulations. In the GBMV calculation, the dielectric constant of water was set to 80 and no distance cutoff was used.

### 2.3. Correlation Analysis

Correlations between all the residues in the different clusters from four systems were analyzed using the normalized covariance to characterize the correlation in motion of protein residues [20,21,22,23], ranging from −1 to 1. If two residues move in the same (opposite) direction in most frames, the motion is considered as (anti-)correlated, and the correlation value is close to −1 or 1. If the correlation value between two residues is close to zero, they are generally uncorrelated. The correlation evaluations were performed using CARMA [24]. The weighted network, optimal/sub-optimal paths in Fab/peptide systems was analyzed using the NetworkView [25] module in VMD (University of Illinois at Urbana–Champaign; http://www.ks.uiuc.edu/Research/vmd/ (accessed on 26 October 2020)).

### 2.4. Calculation of Distance between Transmembrane Domain (TM) Helices

To evaluate the TM distance, the residues on the N- and C-termini were selected: N-terminal Thr652 and C-terminal Ile 673 were selected for the HER2 homodimer; N-terminal Thr652 and C-terminal Ile 673 of HER2 and N-terminal Ile622 and C-terminal Phe643 of EGFR were selected for the EGFR-HER2 heterodimer; N-terminal Thr652 and C-terminal Ile 673 of HER2 and N-terminal Leu634 and C-terminal Val654 of HER3 were selected for the HER3-HER2 heterodimer. The α carbons of above the residues were selected to measure the distance. The distance during the simulation was recorded and averaged, listed in Table 1.

### 2.5. Western Blotting for HER3 Expression and Phosphorylation

SKBR3 cells were obtained from the American Type Culture Collection (ATCC) and cultured in DMEM media (Lonza) containing 10% fetal bovine serum and 1% antibiotics/antimycotics (Invitrogen). Antibodies directed against total HER3 and phosphorylated HER3 were purchased from Cell Signaling Technologies. Trastuzumab was obtained from FDA-Designated pharmacy and heregulin was purchased from Sigma Aldrich. For Western blot analysis, cells were seeded at 5 × 10^5^ in 6-well plates and treated with trastuzumab, heregulin, trastuzumab + heregulin or left untreated. After treatments, whole cell lysate was collected and subjected to Western blotting to detect the phosphorylated and total levels of HER3.

## 3. Results

### 3.1. HER2-Containing Homodimers and Heterodimers with EGFR and HER2 Showed Stable Structures after 500 ns Simulation

**Figure 1:**
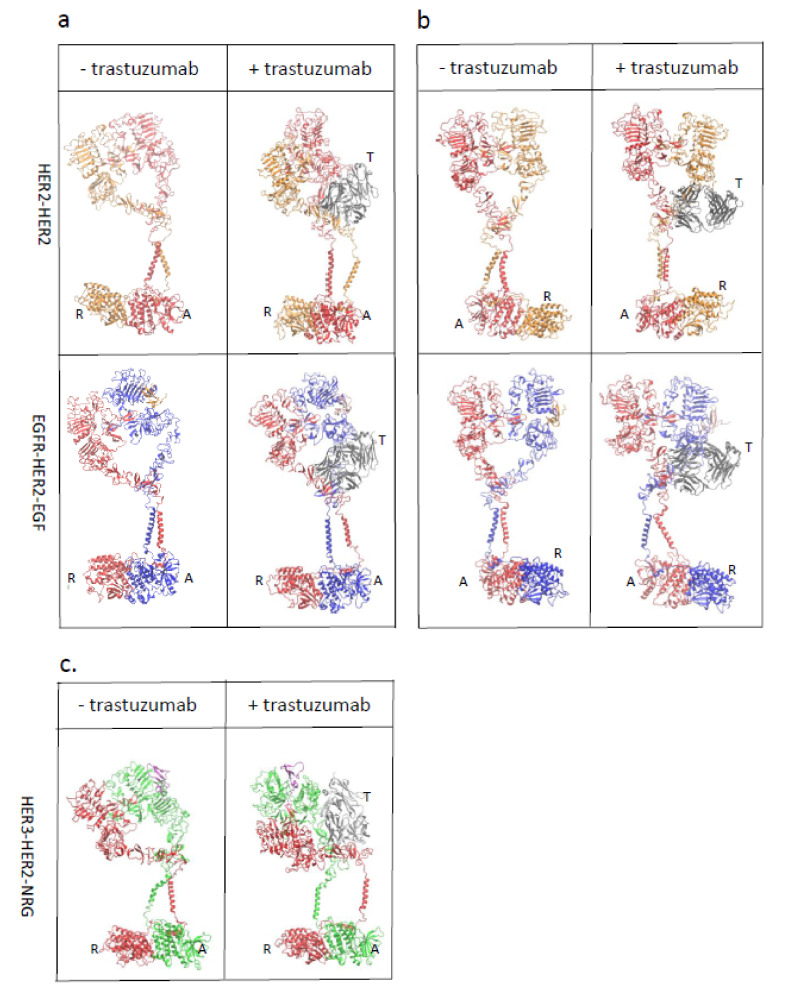
Trastuzumab blocks the receiver function of HER2. The final conformations (after 500 ns simulation) of the HER2-containing homodimers and heterodimers with (right panel) and without (left panel) trastuzumab bound show trastuzumab blocks the receiver function of HER2. HER2 [Receiver (R), gold; Activator (A), red; in the top panels in Figure 1a,b], HER2 (red in the second and third panels in (**a**–**c**)), EGFR (blue in the (**a**,**b**), second panel), HER3 (green in (**c**)), EGF (gold in (**a**,**b**), the second panel in), heregulin (purple in (**c**)), light chain and heavy chain of trastuzumab (T; gray). In the HER2-HER2 homodimer, the two monomers are depicted in yellow (receiver) and red (activator), respectively. Trastuzumab binds to either the receiver, R (**a**) or the activator, A (**b**) conformation of HER2.

The overall structure of EGFR showed symmetry in the extracellular domain and transmembrane helix and asymmetry in tyrosine kinase domain [5]. The asymmetrical structure in tyrosine kinase domain is common among the homodimers and heterodimers of ErbB family proteins [26]. In the asymmetrical structure, one tyrosine kinase domain adopts an activator conformation while the other tyrosine kinase domain adopts a receiver conformation. Based on the structure of the EGFR homodimer, we constructed the HER2-containing homodimers and heterodimers, HER2-HER2 homodimer, EGRF-HER2 and HER3-HER2 heterodimers, by aligning the available crystal structures to the EGFR homodimer (See methods in detail). Two different initial conformations were considered due to the asymmetric structure of the tyrosine kinase domain: HER2 as a receiver in HER2 homo- and heterodimers (Figure 1a, left panels), an activator HER2 homo- and heterodimers (Figure 1b, left panels; Figure 1c, left panel). During the 500-ns simulation, structures of all the dimers remained stable, as shown in the root-mean-square deviation of atomic positions (RMSDs) profiles (Figure S1). The RMSDs of the tyrosine kinase domain of these five dimers were ~6 Å (Figure S1). This suggests that the modeling reached equilibrium and the asymmetrical packing of the tyrosine kinase domain remains stable. A summary of atomistic simulation system of HER2 homo- and hetero-dimers was included in Table S1.

### 3.2. Trastuzumab Blocks the Receiver Function of HER2

The stability of the HER2-containing homodimers and heterodimers was evaluated after trastuzumab bound to HER2. After a 500 ns simulation, two transmembrane helices detached from each other when trastuzumab bound HER2 with a receiver conformation in both HER2-HER2 and EGFR-HER2 dimers (Figure 1a, right panels), whereas the structures appear stable when trastuzumab bound to HER2 with an activator conformation in both HER2-HER2 and EGFR-HER2 dimers (Figure 1b, right panels). These data indicate that trastuzumab, when bound to HER2 containing dimers, blocks the receiver function of HER2.

We measured the distances between the two trans-membrane helices on the N- and C-terminal sides (Table 1). Under the conditions without trastuzumab bound, the distance between the N-termini of HER2 homodimers in the receiver and activator conformations are ~8.5 Å and 8.9 Å, respectively, whereas the distances between the C-termini of HER2 homodimers in the receiver and activator conformations are 22.2 Å and 22.3 Å, respectively. The EGFR-HER2 heterodimer in the HER2 activator conformation showed similar N- and C-terminal distances compared to the HER2 homodimers (Table 1). In contrast, the EGFR-HER2 receiver conformation showed a longer N-terminal distance (11.5Å vs. 8.5 Å) and shorter C-terminal distance (19.9 Å vs. 22.2 Å) as compared to HER2 homodimers (Table 1). The EGFR-HER2 heterodimer in the receiver conformation showed a slight change in the N-(11.5 Å vs. 8.9 Å) and C-terminal distances (19.9 Å vs. 22.3 Å) as compared to that in the HER2 homodimer conformation, while EGFR-HER2 heterodimer in the activator conformation showed essentially no changes in the N-(8.3 Å vs. 8.9 Å) and C-terminal distances (22.7 Å vs. 22.3 Å) as compared to that in the HER2 homodimer conformation. The HER3-HER2 heterodimer in the HER2 activator conformation showed longer N-(13.6 Å vs. 8.9 Å) and C-terminal distances (26.4 Å vs. 22.3 Å) compared to the HER2 homodimers (Table 1).

Under the conditions when trastuzumab bound to HER2 (Table 1), the HER2 homodimer in the activator conformation showed a slight change in the N-(11.9 Å vs. 8.5 Å) and C-terminal (20.8 Å vs. 22.3 Å) distances as compared with HER2 homodimer in the receiver conformation without binding to trastuzumab, whereas the HER2 homodimer in receiver conformation showed a large change in the N-(28.3Å vs. 8.5 Å) and C-terminal distances (27.3 Å vs. 22.2 Å) as compared with trastuzumab free HER2 homodimer in the receiver conformation. The HER3-HER2 heterodimer in the HER2 activator conformation showed a large change in the N-(24.5 Å vs.13.6 Å) and C-terminal distances (43.2 Å vs. 26.4 Å) as compared with the HER3-HER2 heterodimer in the trastuzumab free conformation. These observations suggest when a HER2 monomer adopts an activator conformation, it can form a stable complex with another HER2 monomer or EGFR monomer after trastuzumab binding. This changes the distribution of the conformation population of HER2-containing homodimers and heterodimers. As HER3 can only function as an activator, suggesting that the HER2-HER3 heterodimer function is completely blocked upon trastuzumab binding.

### 3.3. Motion Correlation and Clustering of HER2-Containing Homodimers and Heterodimers before and after Trastuzumab Binding Indicate That Trastuzumab Binding Changes the Flexibility of the Tyrosine Kinase Domain

**Figure 2:**
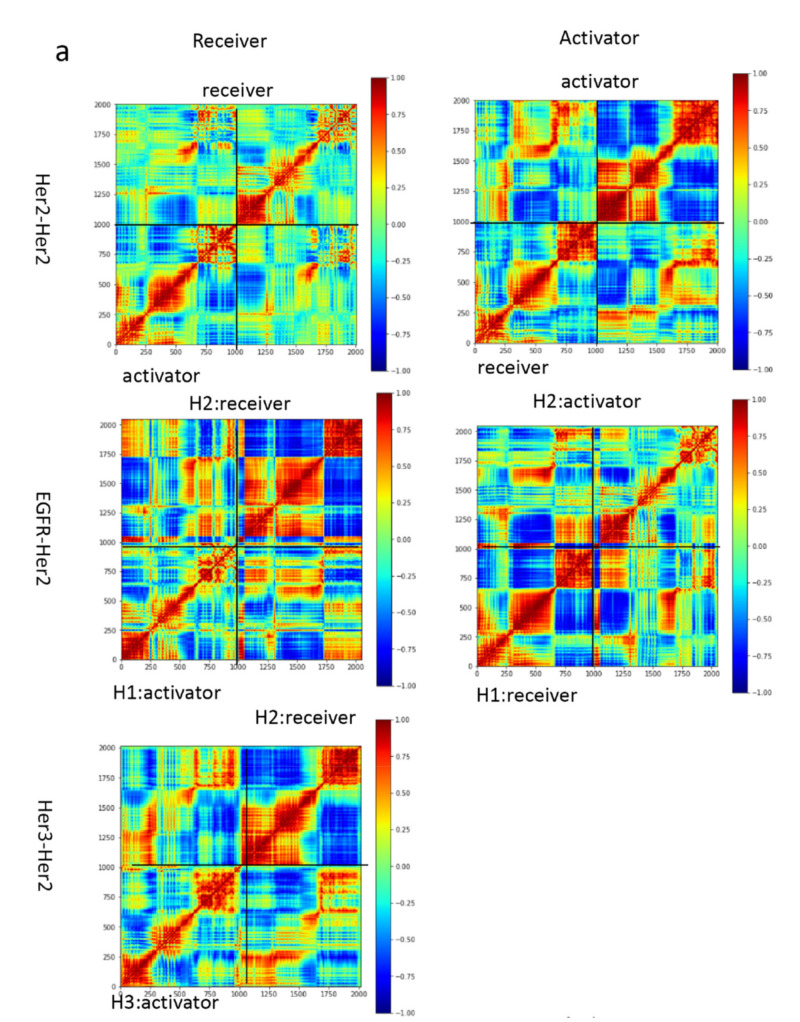

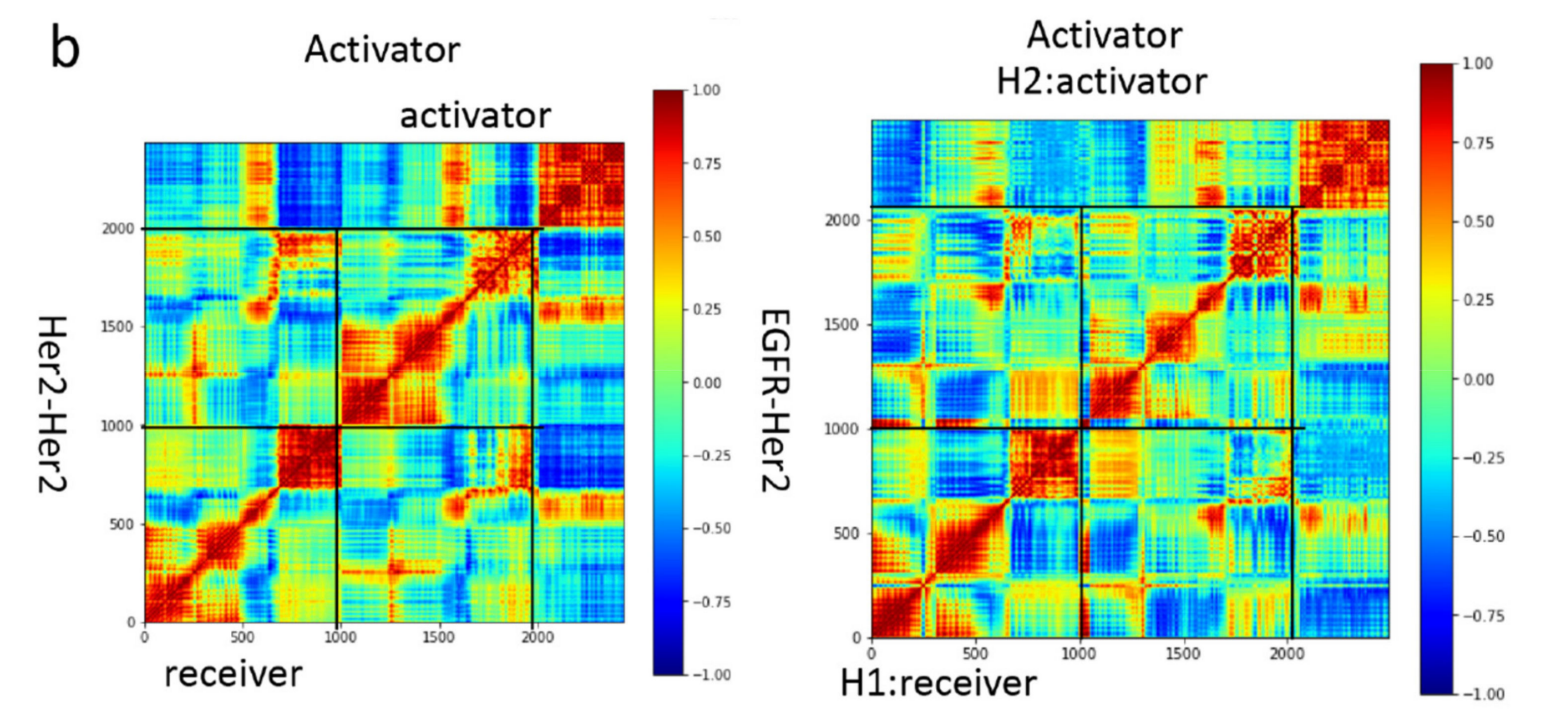
Motion correlation among the residues of the HER2 containing homodimer and heterodimer. Results, (**a**) without trastuzumab bound and (**b**) with trastuzumab bound, indicate that trastuzumab changes the dynamic behavior of the tyrosine kinase domain. The conformation of HER2 can be either activator or receiver listed on the top of the columns. Residues with highly (anti)correlated motion are red (blue).

**Figure 3:**
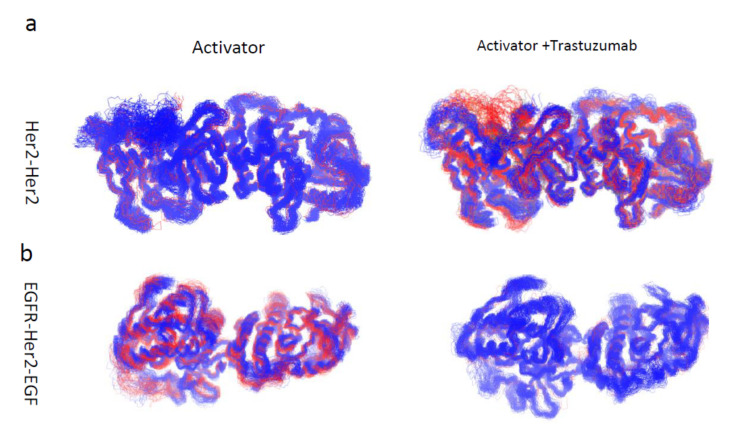
Clustered conformation of the tyrosine kinase domain of the stable HER2-HER2 homodimer (**a**) and EGFR-HER2 heterodimer (**b**) after binding of trastuzumab to homo- and heterodimers. Data obtained were compared to the corresponding apo form and indicated that binding of trastuzumab to the extracellular domain IV of homo- and heterodimers change the conformation of the tyrosine kinase domains. The top three clusters are shown in blue, red, and gray colors, respectively.

The time-correlated protein domain motions and molecular flexibility of the HER2-containing homodimers and heterodimers were analyzed based on the structures obtained from the MD simulation (Figure 2). The blue region indicates the two domains of the protein are moving in opposite direction, while the red region indicated the two domains of the protein were moving in the same direction. The extracellular and the tyrosine kinase domains were moving in opposite directions in the apo form, which decreases after the trastuzumab binding. The tyrosine kinase domain of the HER2-HER2 homodimer and EGFR-HER2 heterodimer, in which HER2 adopts an activator conformation, were analyzed, as these two dimers are stable after binding to trastuzumab (Figure 3). We performed a cluster analysis to evaluate the structural diversity of the tyrosine kinase domain. The HER2-HER2 tyrosine kinase domains are rigid in the apo form and become flexible after binding to trastuzumab (Table 2). In contrast, the EGFR-HER2 tyrosine kinase domains are flexible in apo form and become rigid after trastuzumab binding (Table 2). These results indicate binding of trastuzumab to HER2-containing dimers changes the conformation of the tyrosine kinase domain, which can potentially modify the phosphorylation profiles that are different from ligand-induced phosphorylation profiles in the intracellular domains of EGFR and HER2.

### 3.4. Trastuzumab Reduced HER2 Monomer Flexibility While Pertuzumab Increased HER2 Monomer Flexibility

To evaluate the behavior of the HER2 monomer before and after trastuzumab and/or pertuzumab binding, 300 ns MD simulations were performed. Visual inspection of the trajectories showed the HER2 monomer was highly flexible (Figure S2a). Trastuzumab and pertuzumab Fabs can bind to HER2 extracellular domains IV and II, respectively, at the same time without steric effect. The motion correlation among residues of the HER2 monomer was evaluated in the trastuzumab and/or pertuzumab bound state (Figure S2b). We found trastuzumab reduced and pertuzumab increased the overall HER2 monomer flexibility.

### 3.5. Trastuzumab Reduced HER3 Expression

**Figure 4:**
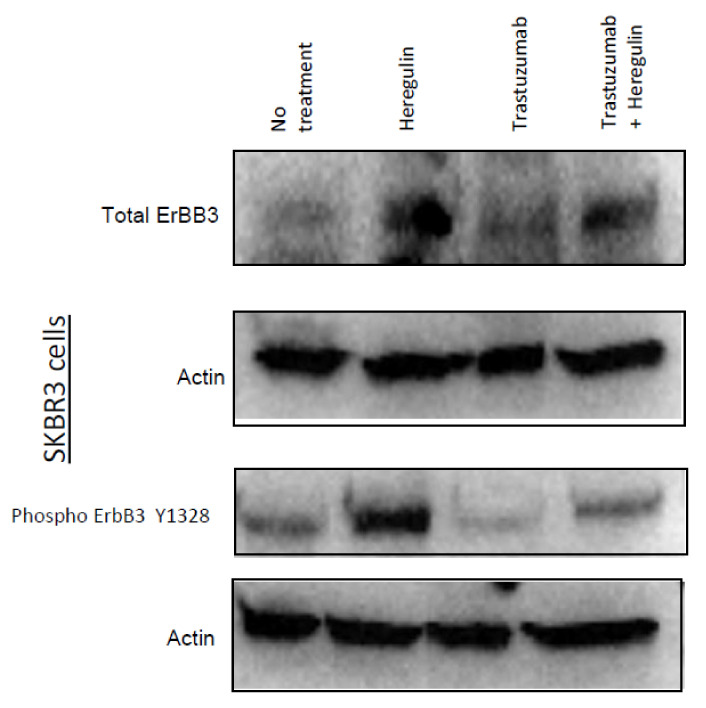
Trastuzumab downregulates heregulin-induced expression and phosphorylation of HER3. SKBR3 cells were seeded in 6-well plates and serum starved overnight. Cells were exposed to heregulin at 100 ng/mL for 15 min, trastuzumab at 4 µg/mL for 1 h, and trastuzumab at 4 µg/mL for 1 h followed by heregulin for 15 min or left untreated. After incubation, whole cell lysate was collected, and Western blotting was performed to monitor the expression and phosphorylation of ErbB3. Actin was used as loading control.

The expression and phosphorylation profiles of ErbB family proteins were evaluated before and after the trastuzumab treatment in SKBR3 cells (Figure 4). Western blotting experiments were performed to assess whether trastuzumab affects heregulin-mediated expression and phosphorylation of HER3. Figure 4 shows that the heregulin drastically enhanced HER3 expression and phosphorylation in SKBR3 cells. Furthermore, heregulin-mediated HER3 phosphorylation was greatly decreased in response to trastuzumab pre-treatment. Trastuzumab destabilized the HER3-HER2 heterodimer, as shown in the MD simulation, therefore, the HER3-HER2 heterodimer population should be reduced due to the unstable structure. This explains the decreased HER3 expression.

## 4. Discussion

### 4.1. Trastuzumab Is Either an Antagonist or a Modulator of HER2 Homo-and Hetero-Dimers

The HER/ErbB family members consist of an extracellular domain, domains I–IV, and an intracellular tyrosine kinase domain with a long C-terminal tail. The extracellular and intracellular domains are connected by a transmembrane helix and a juxtamembrane domain [26]. Without EGF binding, EGFR remains inactive because the intracellular domain remains tethered to plasma membrane and does not dimerize. However, upon EGF binding, the steric constraints are removed at the extracellular domain, which allows the dimerization through N-terminal interactions between transmembrane helices, resulting in EGFR activation. The transmembrane helices favor an N-terminal dimer [5] preferred in EGFR activation [27]. An alternative C-terminal dimer with weak polar interactions is associated with EGFR inactivation [28]. Switching between the two alternative conformations is one of the key stages of HER/ErbB signal transduction. The simulation data suggested that trastuzumab does not favor the dimerization when binding to HER2 receiver. The transmembrane helices dimer cannot maintain the active N-terminal dimer with trastuzumab bound, and disassociate from each other, which shifts the dimer conformation to the inactive C-terminal dimer conformation, leading to the inhibition of activation. In this case, trastuzumab is an antagonist of HER2. In contrast, when trastuzumab binds to HER2 in the activator conformation, the transmembrane helices dimer remains stable and maintains an active N-terminal dimer, thus enabling the trastuzumab to function as an agonist (modulator) of HER2.

It has been reported that a designed peptidomimetic, which specifically binds to HER2 C-terminal domain IV where trastuzumab binds, can block the heterodimerization of EGFR-HER2 and HER2-HER3, leading to the inhibition of phosphorylation of the kinase domain [29]. This suggests that dimerization between the C-termini of domain IV is important for the activation of EGFR-HER2 and HER2-HER3. The trastuzumab binding epitope is close to this dimerization interface; but does not overlap with the interface. Thus, trastuzumab binding does not directly block domain IV dimerization. However, due to asymmetric packing of the tyrosine kinase domains of an activator and a receiver, and the different packing of the juxtamembrane segments from the activator and receiver, there will be different responses to trastuzumab binding. The C-terminal portion of the juxtamembrane segment of the receiver interacts with the activator kinases, and the residues important for this interaction are conserved among the four EGFR family members [27]. These interactions make the receiver more sensitive to trastuzumab binding. The C-terminal portion of the juxtamembrane segment of the activator does not have many intermolecular interactions, and forms a loose coil structure, which tolerates the tension of trastuzumab binding. This potentially explains the trastuzumab binding induced conformation selection.

### 4.2. Heterodimers and Homodimers of HER2 Are Modulated by Trastuzumab

EGF receptors bind different agonist ligands with different affinity, e.g., EGF, TGF, and EPG [30,31], and induce different responses [32,33,34,35]. These ligands usually bind domains I and III, leading to the exposure of domain II for dimerization [3,4,36,37]. HER2 has no known direct activating ligand and is a constitutively active protein. It was reported that HER2 can readily heterodimerize with ligand-activated family members or homodimerize with itself, which makes it the preferred heterodimerization partner of the other family members [38,39,40]. HER3 lacks an active intracellular kinase domain [41], and cannot form homodimers, which makes HER3 participate in hetero-oligomeric assemblies [39]. The simulation results indicated that HER2 can achieve stable conformations of homodimers with itself and heterodimers with EGFR and HER3.

Our data suggest that trastuzumab prevents the receiver conformation of HER2, but not the activator conformation. Thus, trastuzumab cannot completely block the EGFR-HER2 heterodimer. In the HER2-HER3 complex, HER2 functions as a receiver, because HER3 cannot be a receiver due to lack of active intracellular kinase domain. Upon trastuzumab binding, the HER2-HER3 complex becomes unstable because trastuzumab prevents the receiver conformation of HER2. A previous study reported that trastuzumab does not interfere with the interaction between HER2 and HER3 [38]. This biophysical data is inconsistent with our simulation data regarding the stability of HER2/HER3 heterodimer. However, the intracellular domain was not included in that earlier study, which may explain this difference.

### 4.3. The Mechanism of Trastuzumab-Induced Expression and Phosphorylation Changes

**Figure 5:**
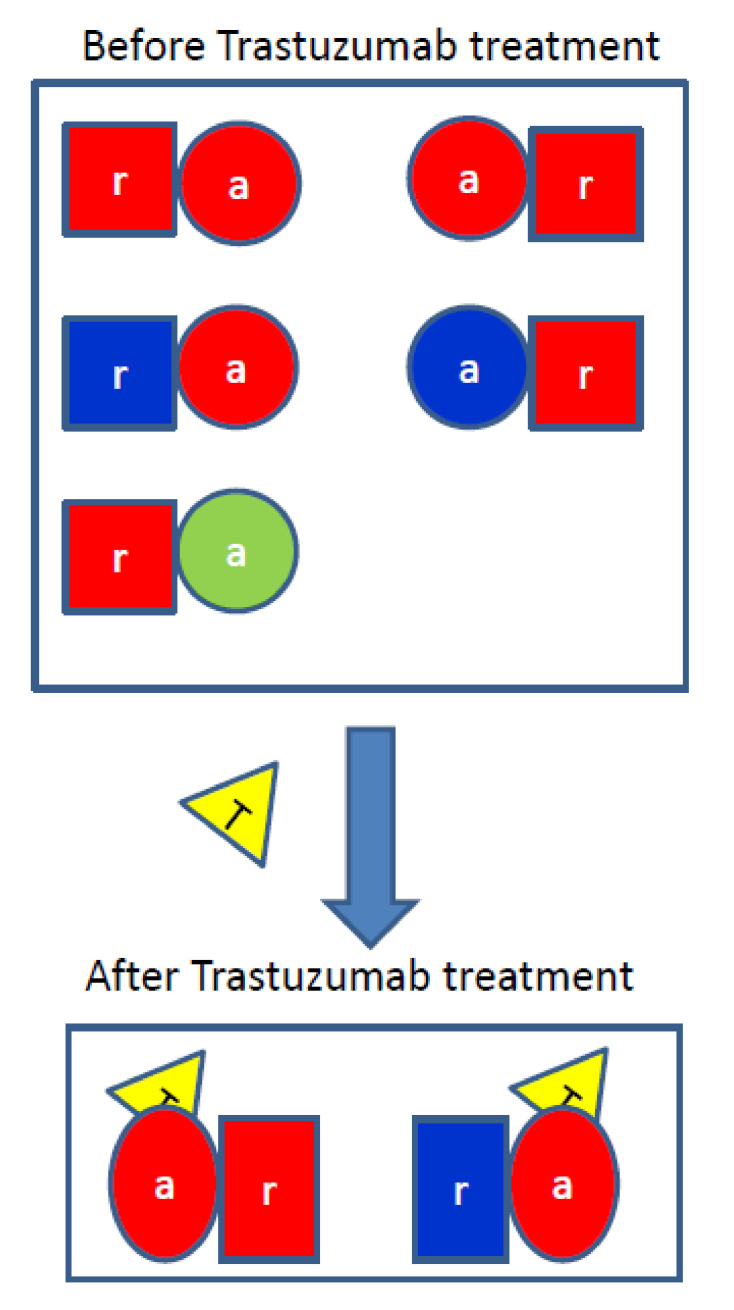
Working model for the mechanism of trastuzumab-induced population shifts of HER2-containing homodimers and heterodimers. The receiver and activator conformations are shown in square and circle, respectively. EGFR (blue), HER2 (red), HER3 (lime), and trastuzumab (yellow).

There are five different HER2-containing dimers population, however only two remain stable after trastuzumab treatment, the HER2 homodimer and EGFR (receiver) HER2 (activator) heterodimer (Figure 5). Moreover, the structures of the tyrosine kinase domains of these two dimers changed after trastuzumab binding. This explains the HER3 downregulation and EGFR/HER2 phosphorylation change. Our previous data showed trastuzumab treatment did not reduce EGFR or HER2 expression, but changed the phosphorylation profile of EGFR and HER2, i.e., ErbB1-pY845 and ErbB2-pY1248 [42,43]. Several studies have demonstrated there is significant conformational and functional linkage between the EGFR extracellular and intracellular domains, and these conformations remain in equilibrium at the steady state [44,45]. Small molecule tyrosine kinase inhibitors of EGFR, which function as allosteric regulators, shift the conformational equilibrium between active and inactive species of EGFR, suggesting there is strong functional coupling between the extracellular and intracellular domains [44]. The monoclonal antibodies cause allosteric long-range conformational changes on their antigen binding partner [46,47]. These allosteric effects induce the change of the biological properties and enhance binding of antibodies to the antigen, or negative allosteric effects. A series of monospecific F(ab’)2-like molecules of trastuzumab were produced by introducing cysteine substitutions into various positions in the heavy and light chains of Fab region of trastuzumab and these isomer molecules demonstrated various activities ranging from activation to inhibition of breast tumor cell growth [48]. Quantitative phosphorylation mapping of HER2 indicates the agonistic isomers produced a distinct phosphorylation pattern associated with activation. Our biochemical data suggested that trastuzumab Fab binding to domain IV can induce changes in the expression and phosphorylation profile of HER2. Trastuzumab binding shifts the conformation of the assembly either toward the HER2-containing homodimer or the HER2-EGFR heterodimer.

In this work, the unstructured long C-terminal tails were not included as these structures have not been determined. Although some structural models have been proposed for the C-terminal tails [49], the long (>200 amino acids) and dynamic nature of the tail make it challenging to model the whole structure of EGFR. It is expected that the C-terminal tail may shift the conformation, leading to changes in the phosphorylation profile, as discovered in this work.

## Figures and Tables

**Table 1:** Distance between transmembrane helices.

HER Dimers	Ligand	Conformation	TrastuzumabBound	Distance between N-Termini (Å)	Distance between C-Termini (Å)
HER2+HER2	n/a	receiver	no	8.5 ± 0.5	22.2 ± 2.4
HER2+HER2	n/a	activator	no	8.9 ± 0.5	22.3 ± 1.5
HER2+HER2	n/a	receiver	yes	28.3 ± 6.8	27.3 ± 4.6
HER2+HER2	n/a	activator	yes	11.9 ± 1.3	20.8 ± 2
HER1+HER2	yes	receiver	no	11.5 ± 1.2	19.9 ± 1.5
HER1+HER2	yes	activator	no	8.3 ± 1	22.7 ± 1.8
HER1+HER2	yes	receiver	yes	13.1 ± 3.7	24.9 ± 2.4
HER1+HER2	yes	activator	yes	11.9 ± 2.3	28.8 ± 2.5
HER3+HER2	yes	activator	no	13.6 ± 1.7	26.4 ± 2.1
HER3+HER2	yes	activator	yes	24.5 ± 7.5	43.2 ± 3.6

**Table 2:** The top three clustered conformation of the tyrosine kinase domain of the stable homodimer/heterodimer after trastuzumab binding compared with the corresponding apo form.

	− Trastuzumab	+ Trastuzumab
HER2-HER2	93/6/1	59/31/5
EGFR-HER2	61/36/3	94/32/1

## Data Availability

Not applicable.

## Supplementary Materials

The following are available online at https://www.mdpi.com/2073-4468/10/1/7/s1, Figure S1. RMSDs of the tyrosine kinase domain dimers during the 500 ns simulation. HER2 homodimers (blue and red); EGFR-HER2 heterodimers (gray and orange); HER3-HER2 heterodimers (yellow). Figure S2. Trastuzumab reduces and pertuzumab increases HER2 monomer flexibility. (a) The conformation of the HER2 monomer in complex with trastuzumab, pertuzumab, and combination of trastuzumab and pertuzumab; extracellular domain (red), transmembrane helix (lime), tyrosine kinase domain of HER2 (cyan), trastuzumab (yellow), and pertuzumab (purple). (b) Motion correlation among the residues of the HER2 monomer. Residues with highly correlated motion (red) and anticorrelated motion (blue); Table S1. Summary of atomistic simulation systems of HER2 homodimers and heterodimers.
