# Supplementary material for: Trastuzumab Blocks the Receiver Function of HER2 Leading to the Population Shifts of HER2-Containing Homodimers and Heterodimers
Source: Antibodies (Basel). 2021 Feb 4;10(1):7. doi: 10.3390/antib10010007 (PMC7931022; doi:10.3390/antib10010007)
Supplement: Supplementary file 1 [file antibodies-10-00007-s001.zip › antibodes-1000986-supple/antibodies-1000986-supple.docx]

**Table S1.** Summary of atomistic simulation systems of HER2 homodimers and heterodimers.

| **HER dimers** | **Ligand** | **Trastuzumab Binding** | **Conformation of HER2** | **Time (ns)** |
| --- | --- | --- | --- | --- |
| HER2+HER2 | n/a | no | receiver | 500 |
| HER2+HER2 | n/a | no | activator | 500 |
| HER2+HER2 | n/a | yes | receiver | 300 |
| HER2+HER2 | n/a | yes | activator | 500 |
| HER1+HER2 | yes | no | receiver | 500 |
| HER1+HER2 | yes | no | activator | 500 |
| HER1+HER2 | yes | yes | receiver | 300 |
| HER1+HER2 | yes | yes | activator | 500 |
| HER3+HER2 | yes | no | receiver | 500 |
| HER3+HER2 | yes | yes | receiver | 300 |
