# Supplementary figures and images for: Trastuzumab Blocks the Receiver Function of HER2 Leading to the Population Shifts of HER2-Containing Homodimers and Heterodimers
Source: Antibodies (Basel). 2021 Feb 4;10(1):7. doi: 10.3390/antib10010007 (PMC7931022; doi:10.3390/antib10010007)

Fig. S1

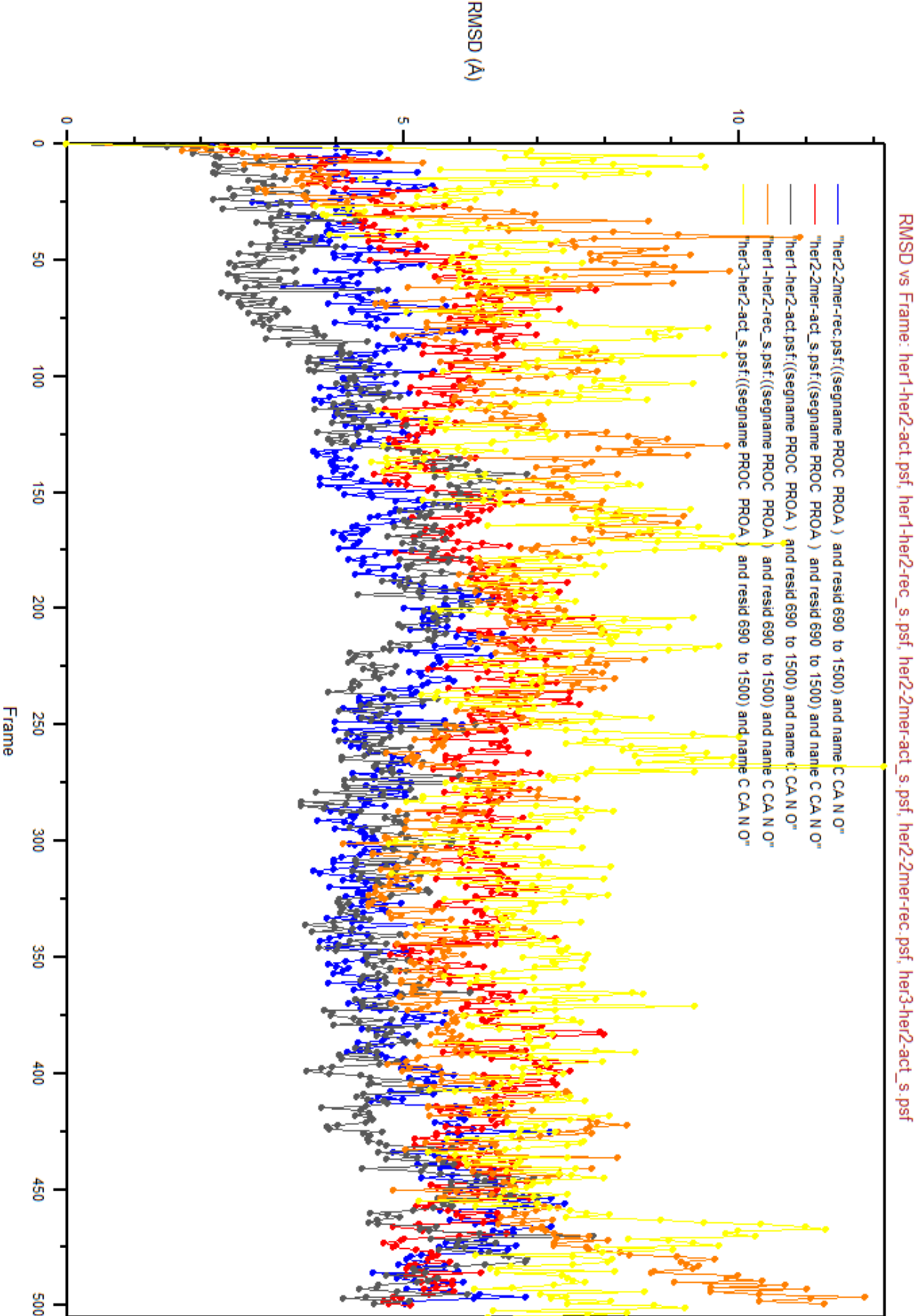

Fig. S2

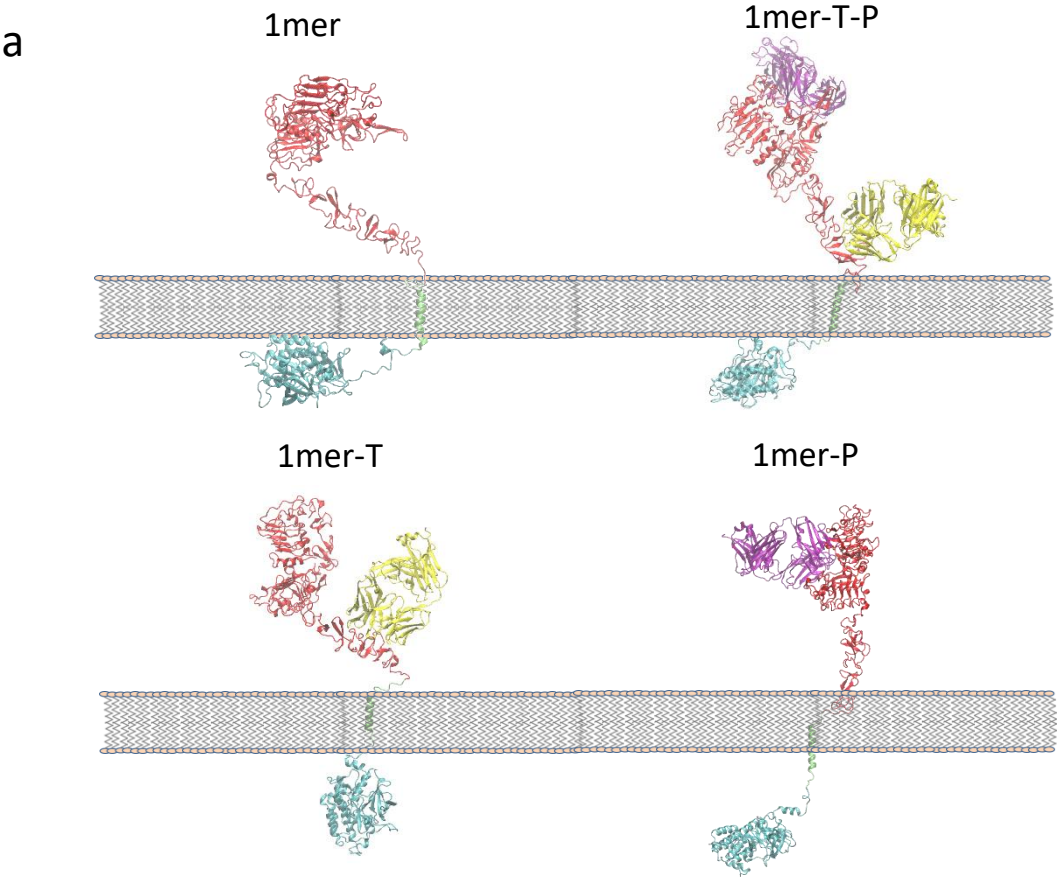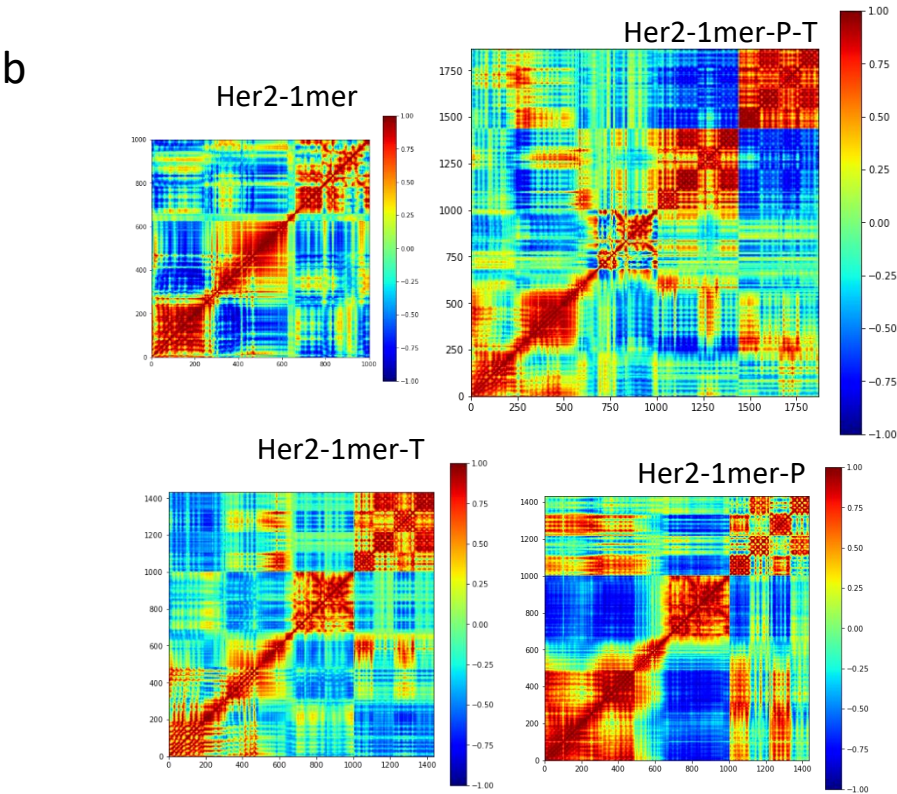

Supplement: Supplementary file 1 [file antibodies-10-00007-s001.zip › antibodes-1000986-supple/antibodies-1000986-supplementary.pdf]
